# Antibacterial activity of extracellular compounds produced by a *Pseudomonas* strain against methicillin-resistant *Staphylococcus aureus* (MRSA) strains

**DOI:** 10.1186/1476-0711-12-12

**Published:** 2013-06-17

**Authors:** Viviane F Cardozo, Admilton G Oliveira, Erick K Nishio, Marcia RE Perugini, Célia GTJ Andrade, Wanderley D Silveira, Nelson Durán, Galdino Andrade, Renata KT Kobayashi, Gerson Nakazato

**Affiliations:** 1 Department of Microbiology, Biology Sciences Center, University of Londrina State, Londrina, PR CP 86005-990, Brazil; 2 Department of Pathology, Clinical Analysis and Toxicological, University of Londrina State, Londrina, PR CP 86038-440, Brazil; 3 Department of General Biology, University of Londrina State, Londrina, PR CP 86051-990, Brazil; 4 Department of Genetics, Evolution and Bioagent, Biology Institute, Campinas State University (UNICAMP), Campinas, SP CP 13083-970, Brazil; 5 Department of Physical Chemistry, Chemistry Institute Campinas State University (UNICAMP), Campinas, SP CP 13083-970, Brazil

**Keywords:** Antibacterial activity, Methicillin-resistant, *Pseudomonas aeruginosa*, *Staphylococcus aureus*

## Abstract

**Background:**

The emergence of multidrug-resistant bacteria is a world health problem. *Staphylococcus aureus,* including methicillin-resistant *S. aureus* (MRSA) strains, is one of the most important human pathogens associated with hospital and community-acquired infections. The aim of this work was to evaluate the antibacterial activity of a *Pseudomonas aeruginosa*-derived compound against MRSA strains.

**Methods:**

Thirty clinical MRSA strains were isolated, and three standard MRSA strains were evaluated. The extracellular compounds were purified by vacuum liquid chromatography. Evaluation of antibacterial activity was performed by agar diffusion technique, determination of the minimal inhibitory concentration, curve of growth and viability and scanning electron microscopy. Interaction of an extracellular compound with silver nanoparticle was studied to evaluate antibacterial effect.

**Results:**

The F3 (ethyl acetate) and F3d (dichloromethane- ethyl acetate) fractions demonstrated antibacterial activity against the MRSA strains. Phenazine-1-carboxamide was identified and purified from the F3d fraction and demonstrated slight antibacterial activity against MRSA, and synergic effect when combined with silver nanoparticles produced by *Fusarium oxysporum*. Organohalogen compound was purified from this fraction showing high antibacterial effect. Using scanning electron microscopy, we show that the F3d fraction caused morphological changes to the cell wall of the MRSA strains.

**Conclusions:**

These results suggest that *P. aeruginosa*-produced compounds such as phenazines have inhibitory effects against MRSA and may be a good alternative treatment to control infections caused by MRSA.

## Introduction

The emergence of multidrug-resistant bacteria is a world health problem [1,2]. *Staphylococcus aureus* is one of the most important human pathogens associated with hospital and community-acquired infections. Over the last few decades, the number and proportion of methicillin-resistant *S. aureus* (MRSA) infections in different countries has increased due to the rise of epidemics in humans [3-5] and other animals, such as dogs, cats, cattle, pigs and exotic species [6,7]. In Brazil, according to data obtained from the first five years of the SENTRY Antimicrobial Surveillance Program, MRSA strains were among the most prevalent pathogens and contributed to 56% of the nosocomial and community infections [8]. One of the major global clones is the MRSA Brazilian epidemic clone (BEC), a hospital-acquired MRSA strain. Isolates of this strain are typically resistant to multiple antimicrobials [9].

The expense incurred to control MRSA may be considerable; however, several economic evaluations have indicated that MRSA control programs are cost-effective in terms of reducing the costs of MRSA infections. In a study comparing two neonatal ICUs, the cost of instituting control measures in a stepwise, delayed approach was US$ 49–69 million (€ 38–52 million), while the cost of introducing effective and immediate measures was US$ 1.3 million (€ 1 million) [10]. Another study calculated that the total cost per case of bacteremia that was caused by an antibiotic-resistant strain, including MRSA (50% of the cases), was US$ 88,445 [11].

The health risks associated with MRSA infections, including its potential to produce invasive infections, particularly in vulnerable patients, and its resistance to multiple antibiotics, warrant the implementation of monitoring programs to control its dissemination. There is a considerable epidemiological interest in tracking strains to gain a more complete picture of the distribution of strains in the population and the dynamics of clonal spread [12]. For years, vancomycin has been used as the drug of choice to treat MRSA infections and was recommended by clinical guidelines; however, the emergence of the vancomycin-resistant *S. aureus* (VRSA) and vancomycin-intermediate *S. aureus* (VISA) has made antibacterial therapy difficult. Therefore, new chemotherapeutic compounds to treat and control infections by these microorganisms have been broadly studied and developed [13].

Recently, some natural antibacterial agents, such *Quercus dilatata*, *Hylomecon hylomeconoides, Eleutherine Americana*, *Chelidonium majus* Linn. (*Papaveraceae*) and *Tabebuia avellanedae* compounds, have been tested against MRSA [14-18].

The ability of antibacterial compounds obtained from other bacteria to inhibit methicillin-sensitive *S. aureus* (MSSA) and MRSA has also been tested [19-21]. Other bacterial compounds known to have antibacterial activity have not been tested against MRSA. We have tested an extracellular compound derived from *Pseudomonas aeruginosa* that has previously been shown to have antibacterial effects against *Xanthomonas citri* pv. Citri, which causes citrus cancer lesions [22].

The aim of this work was to evaluate the antibacterial activity of a compound from *P. aeruginosa* against MRSA strains.

## Materials and methods

### Bacteria strains

Thirty MRSA strains from bacteria collection of the hospital of Londrina State University, and isolated in 2011, Londrina-PR, Brazil. The MRSA strains were isolated from blood, urine, trachea and secretion cultures. Three standard MRSA strains were also used in this work. The strains MRSA N315 [23], BEC9393 [24] and rib1 [25] were provided by Dr. Elsa Masae Mamizuka (Universidade de São Paulo, São Paulo-SP, Brazil), Dr. Agnes Marie Sá Figueiredo (Universidade Federal do Rio de Janeiro, Rio de Janeiro-RJ, Brazil), and Dr. Wanderley Dias da Silveira (Universidade Estadual de Campinas, Campinas-SP, Brazil), respectively. All strains were stored at - 80°C in stocks containing glycerol (2.5 M).

### Extracellular compounds from *Pseudomonas aeruginosa*

The extracellular compounds were provided by Dr. Galdino Andrade from the Laboratory of Microbial Ecology (Londrina State University, Londrina-PR, Brazil). The method of production has been patented (Patent, 2008#PI0803350-1; http://www.inpi.gov.br). These antibacterial compounds were obtained from the *P. aeruginosa* LV strain that was isolated from an old citrus canker lesion on the leaves of orange (*Citrus sinensis* cv. Valence) plants and collected in Astorga, Brazil [26]. The production and purification of these compounds by vacuum liquid chromatography (VLC) were performed as described by Oliveira and collaborators (2011) [22]. The culture supernatants were treated with dichloromethane 1:1 (v:v). The dichloromethane phase (DP) was fractionated using the mobile phase (v/v): hexane (100:F1); dichloromethane (100; F2); ethyl acetate (100; F3); methanol (100; F4); methanol–water (1:1; F5); and water (100; F6). Fractionation was performed again using the following phase (v/v): hexane (100; F3a); hexane-dichloromethane (1:1; F3b); dichloromethane (100; F3c); dichloromethane- ethyl acetate (1:1; F3d); ethyl acetate (100; F3e); ethyl acetate-methanol (1:1; F3f); methanol (100; F3g); methanol–water (1:1; F3h); and water (100; F3i). In this study, the F3 and F3d fractions were used to evaluate the antibiosis effect. All reagents were purchased from Sigma-Aldrich, USA.

### Silver nanoparticles from *Fusarium oxysporum*

The silver nanoparticles were obtained following the method of Durán and collaborators (2005) [27]. After the growth of *F. oxysporum* culture, 10 g of the biomass was added in 100 ml of distilled water. After incubation of 72 h at 28°C, the solution components were separated by filtration, and AgNO3 at concentration of 10^-3^M was added and the system was kept for several hours and then the absorbance at 420 nm that corresponds to the plasmon resonance value was determined. The silver nanoparticles were characterized by Transmission Electron Microscopy (TEM) (Carl Zeiss CEM-902, 80 KeV).

### Cytotoxicity assay

The LLC-MK_2_ cell line was cultured in a 96-well culture plate at a density of 2.5 × 10^5^ cells/well and incubated for 24 h. When the cells were confluent, the non-adherent cells were removed by washing with sterile 0.01 M phosphate buffered saline (PBS). The medium containing different concentrations of F3d (1 to 2000 *μ*g/ml) was added to each well containing the cells, and the plates were incubated for 72 h. For the controls, the cells were cultured in the growth medium alone or in the presence of 1% dimethyl sulfoxide (DMSO). Cell viability was determined by the dimethylthiazol diphenyl tetrazolium bromide (MTT, Sigma-Aldrich, USA) method, according to the manufacturer’s recommendation. The concentration of the compounds needed to inhibit the viability of cells by 50% (IC_50_) was determined by regression analysis. The 50% cytotoxic concentration (CC_50/72h_) and the selectivity index (SI) were calculated using the equation: SI = CC_50_/IC_50_.

### Evaluation of the antibiosis effect by the agar diffusion technique

The experiment was carried out with three replicates of two fractions obtained from the dichloromethane phase at two concentrations (100 and 500 μg/ml). The negative control was the dichloromethane phase alone (compound solvent). The antibiotic effect of the fraction on the MRSA strains was evaluated on Mueller Hinton agar plates (Difco, USA). MRSA suspensions of 10^8^ colony-forming units (CFU)/ml were grown to log phase, and the diffusion disks were treated with the antibiotic compounds. The plates were incubated at 35°C for 24 h, and the size of the inhibition halos diameter was evaluated (mm). The experiment was repeated three times, and the antibiosis effect was determined by measuring the size of inhibited halos formed around the wells.

### Determination of the minimal inhibitory concentration (MIC)

The minimal inhibitory concentrations (MICs) were determined by micro-dilution assays in 96-well plates, as suggested by the CLSI (2011) [28]. In brief, single colonies of bacterial cultures grown in Mueller-Hinton agar (Sigma-Aldrich, USA) media were diluted in saline solution and adjusted to 0.5 on the MacFarland scale, which corresponds to 1.5 × 10^8^ CFU/ml. Then, the bacterial suspensions were diluted in Mueller-Hinton broth (Difco, USA) and plated in 96-well plates at a density of 5.0 × 10^5^ CFU/well. Finally, different concentrations of the analyzed antibiotics and compounds were added to each well to determine the MIC values. As negative control, DMSO (Sigma-Aldrich, USA) alone was added in equal concentrations as the ones used to solubilize the antibiotic compounds. The plates were incubated at 37°C for 18 h, and then the optical density values at 600 nm were determined using a Bio-Rad Microplate Reader model 3550.

### Curve of growth and viability

To quantify the effect of compounds on the bacterial growth, a time-response growth curve was obtained in the presence of these antimicrobials. In brief, a single colony forming unit (CFU) of each MRSA strain was diluted in Mueller-Hinton broth and grown for 18 hours at 37°C with constant stirring at 200 rpm. Then, each culture was adjusted to 0.5 index in MacFarland scale and inoculated at a cell density of 10^6^ CFU/ml in 2 ml of Mueller-Hinton broth. For each strain culture was divided in two new cultures of 1 ml each. One culture received the antimicrobial compound and other received only the solvent (control). The bacterial cultures were then incubated at 37°C with constant stirring (200 rpm). In different times, an aliquot of the broth was collected, serial diluted in saline solution, plated on Mueller-Hinton agar media and grown for 18 h at 37°C in order to determine the total CFU of each culture.

### Drug interaction studies

To evaluate the antibacterial effects and interactions of phenazine-1-carboxamide combined with silver nanoparticle produced by *Fusarium oxyporum* against MRSA, assays of microdilution in double-antimicrobial gradient were used. Briefly, the MIC values for phenazine and silver nanoparticle used alone were determined, and several concentrations of phenazine were combined with different concentrations of the silver nanoparticle. The MIC of the combination, which is the lowest concentration of phenazine that, when combined with the lowest concentration of silver nanoparticle, were determined. To evaluate the interaction between both antimicrobials, the fractionated inhibitory concentration (FIC) index was used as described by Chin and collaborators (1998) [29]:

$$\mathrm{FIC}=\mathrm{MIC}\left(\mathrm{Pc}\right)/\mathrm{MIC}\left(\mathrm{Pa}\right)+\mathrm{MIC}\left(\mathrm{Sc}\right)/\mathrm{MIC}\left(\mathrm{Sa}\right)$$

Where MIC(Pc) is the MIC of phenazine used combined with the silver nanoparticle, MIC(Pa) is the MIC of free phenzine used alone, MIC(Sc) is the MIC of the silver nanoparticle used combined with phenazine and MIC(Aa) is the MIC of the silver nanoparticle used alone. FIC indexes were interpreted as follows: FIC ≤ 0.5 = synergic interaction; 0.5 < FIC ≤ 1.0 = additive interaction; 1.0 < FIC ≤ 4.0 = no interaction; FIC > 4.0 = antagonist interaction.

### Scanning Electron Microscopy (SEM)

For scanning electron microscopy (SEM), suspensions of the MRSA strains (10^10^ CFU/ml) with and without the antibacterial compound (at about MIC) were spotted onto polylysine-coated glass slides. Each slide was then fixed by immersion in 1 ml of 2.5% glutaraldehyde and 2% paraformaldehyde in 0.1 M sodium cacodylate buffer (pH 7.2) for 12 h and then post-fixed in 1% OsO_4_ for 2 h. The fixed samples were dehydrated in an ethanol gradient (70, 80, 90 and 100˚GL) and then were critical point dried in CO_2_ (BALTEC CPD 030 Critical Point Dryer). Finally, the slides were taped onto stubs, coated with gold (BALTEC SDC 050 Sputter Coater) and observed under a FEI Quanta 200 SEM. All reagents were purchased from Sigma-Aldrich, USA.

## Results

### Extracellular compound

Phenazine-1-carboxamide and organohalogen were identified from the F3d fraction. These compounds were extracted, purified, and evaluated for antibiosis effects. The organohalogen specific structure does not identified yet.

### Cytotoxicity assay

It was not possible to determine the 50% cytotoxic concentration of the F3d fraction on LLC-MK_2_ cells because even with the highest concentration tested (2000 *μ*g/ml), 84% of the cells were viable, according to the MTT assay.

### Diffusion disc-mediated antibiotic treatments against MRSA strains

As an initial screen to evaluate the antimicrobial activity again MRSA, we measured the diameters of the zone of inhibition generated by the F3 fraction against the MRSA standard and clinical isolates (Table 1). There was no difference in the size of the zones for the MRSA standards; however, there was some variation in the zones for the clinical isolates. Discs treated with only dichloromethane (solvent) were also tested and showed no inhibition zones. Discs were impregnated with 25 μg of organohalogen from F3d fraction showed high zones of inhibition for MRSA strain N315 (Table 1). The strains MRSA N315, BEC9393 and rib1 showed resistance for erythromycin, gentamicin, penicillin and ampicillin antimicrobials (data not shown).

**Table 1:** The MIC and diameters of the zones of inhibition (mm) generated by the F3 fraction diffusion discs against standard MRSA strains grown on agar

**MRSA strains**	**F3 disk (500 μg) zone (mm)**	**F3 disk (100 μg) zone (mm)**	**Organohalogen (25 μg) zone (mm)**	**MIC F3 (μg/ml)**	**MIC F3d (μg/ml)**	**MIC phenazine (μg/ml)**
N315	22	12	28	125	125	250
BEC9393	23	12	NT	125	125	NT
Rib1	22	12	NT	125	NT	NT
MRSA clinical	16 - 27	08 - 15	NT	NT	NT	NT

*NT* not tested.

*MIC* Minimal Inhibitory Concentration.

*MRSA* Methicillin-resistant *Staphylococcus aureus.*

### Minimal Inhibitory Concentration (MIC)

The MICs of the F3 fraction for MRSA strains (N315, BEC9393 and Rib1) were in the range of 125 μg/ml. The MIC for the more purified F3d fraction was equal to the F3 fraction. The MIC for phenazine-1-carboxamide was 250 μg/ml for the MRSA strain N315 (Table 1). These compounds do not have breakpoints because they are new antibiotics (Table 1). The MRSA strain N315 was further selected for curve of growth and viability tests and scanning electron microscopy.

### Curve of growth and viability

**Figure 1:**
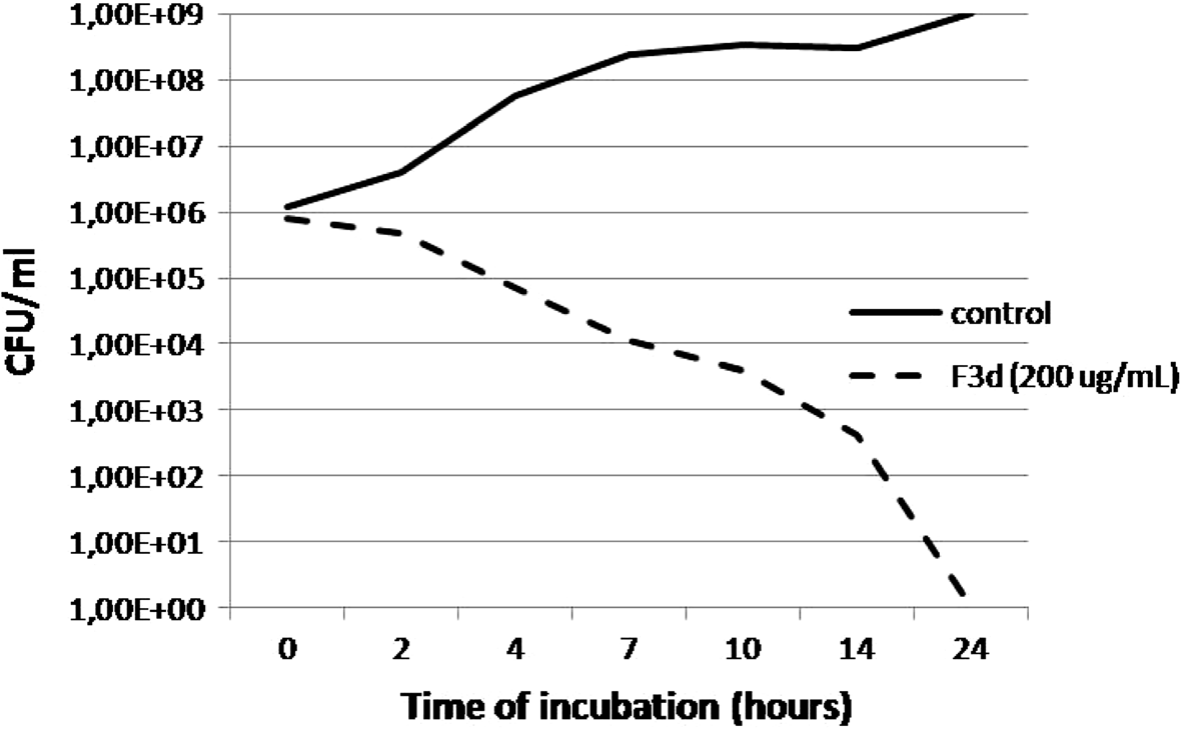
**Time-kill curves of *Staphylococcus aureus *N315 strain exposed to F3d fraction Notes - straight line: without antibiotic treatment; dash line: F3d treatment (200 μg/ml).**

**Figure 2:**
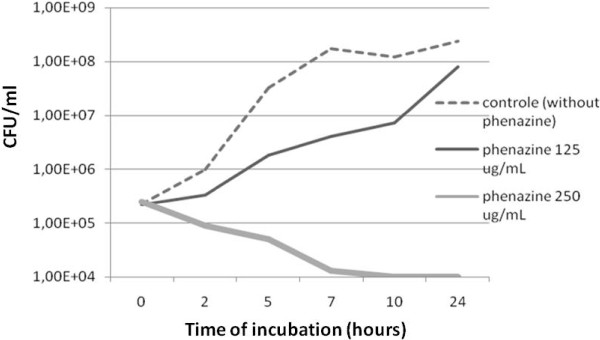
**Time-kill curves of *Staphylococcus aureus *N315 strain exposed to phenazine-1-carboxamide.** Notes - dash line: control; straight line: phenazine treatment (125 μg/ml); large line: phenazine treatment (250 μg/ml).

At 125 μg/ml, the F3 fraction significantly reduced the number of CFUs over the incubation time (data not shown). A similar effect occurred with the F3d fraction (200 μg/ml) (Figure 1) and with phenazine-1-carboxamide (250 μg/ml) (Figure 2). The number of CFUs after 7 h of incubation with the F3d fraction (200 μg/ml) and phenazine-1-carboxamide (250 μg/ml) was about 10,000-fold lower than the control (without treatment) (Figures 1 and 2). After 24 h of incubation with the F3d fraction, all the bacteria were eliminated (Figure 1).

### Drug interaction studies

Phenazine-1-carboxamide was combined with silver nanoparticle for evaluation synergic effect against MRSA N315 strain. The MICs for phenazine and nanoparticle alone were 250 μg/ml and 125 μM respectively. The MICs for these compounds combined were 7.81 μg/ml and 31.25 μM, showing the FIC at 0.281 (synergic interaction).

### Scanning Electron Microscopy (SEM)

**Figure 3:**
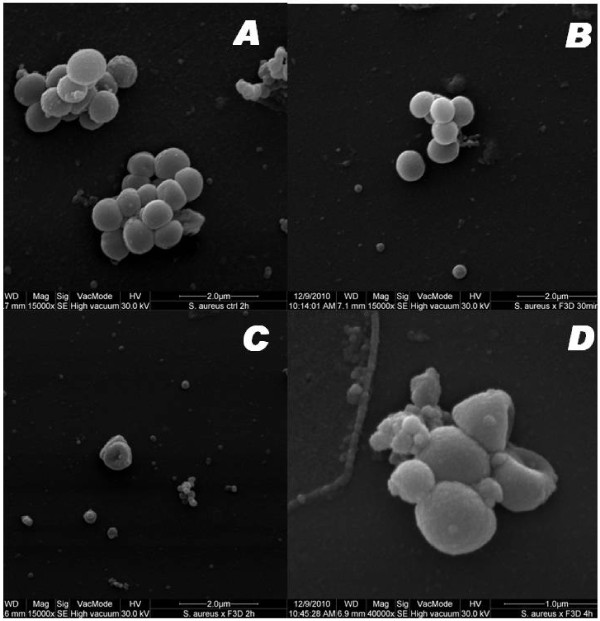
**Scanning electron microscopy images of the antibacterial effect of the F3d fraction (200 μg/ml) against the MRSA N315 strain at different times. A**: negative control (2 h without antibiotic); **B**: F3d (30 min); **C**: F3d (2 h); **D**: F3d (4 h). When the bacteria were incubated with the F3d fraction for 2 and 4 h, morphological alterations were observed. No morphological cellular alterations were observed with 30 min incubation.

The SEM analysis showed that at a low concentration of F3d, morphological changes in the bacteria could be observed within a few hours (Figure 3C and 3D). No morphological changes were observed after 30 min of incubation with F3d, but the number of cells was reduced (Figure 3B). After 2 h, we observed the cell wall sinking into the bacterial body (Figure 3C). Some cells were deformed after 4 h (Figure 3D). In contrast, the untreated cells (treated only with solvent) appeared intact, and the cell wall was not deformed (Figure 3A). This assay was performed with 100 μg/ml of the F3d fraction.

## Discussion

The continuous selection of bacteria that are resistant against a wide range of antibiotics necessitates the discovery of novel unconventional sources of antibiotics, mainly in hospitals. Methicillin-resistant *S. aureus* (MRSA), *Escherichia coli* O157:H7, *Mycobacterium tuberculosis* and *P. aeruginosa* have been considered some of the most virulent microorganisms for the human population.

Notably, MRSA and VISA strains have a thickened cell wall that is believed to deplete the vancomycin available to kill the bacterium; this mechanism of resistance would significantly impact the near future prospects of the current anti-MRSA therapies. The methanol crude extract of *Brassica oleracea* L. (red cabbage) was investigated for possible antimicrobial activity. The anti-MRSA activity of the red cabbage extract and its underlying mechanism of action appear to be novel and different from other known antibiotics. Accordingly, the discovery of natural, effective, and cheap drugs against this resistant bacterium may be a breakthrough solution for this worldwide problem [30].

The pentacyclic triterpenoids α-amyrin, betulinic acid and betulinaldehyde and other related triterpenes, such as imberbic acid, oleanolic acid (oleanic acid), ursolic acid, ulsolic acid, rotundic acid and zeylasteral, have been reported to possess antimicrobial activity [31]. Preliminary studies have shown that the pentacyclic triterpenoids have weak antibacterial activity against the reference strains of methicillin-resistant (ATCC 43300) [31]. All three triterpenoids exhibited a bacteriostatic effect against the reference strains of *S. aureus* at the concentrations tested. Synergism against the two reference strains was reproducibly observed between the three compounds and cell wall inhibitors of the β-lactam and glycopeptide classes. The best synergistic combination was betulinic acid and methicillin [31].

The bactericidal effect of the ethanolic extracts of the stem bark of cinnamon (*Cinnamomum zeylanicum*; CIN), the flower bud and stalk of clove (*Syzygium aromaticum*, CLV) and the seed of cumin (*Cuminum cyminum*, CMN) were tested on MRSA. In decreasing order, the antibacterial activities for the spices were *C. zeylanicum* > *S. aromaticum* > *C. cyminum*. All three spices were excellent bactericidal agents and are potential anti-MRSA agents [32].

Thiomarinols, produced by marine bacteria belonging to the genus *Pseudoalteromonas*, are hybrids of two independently active species: the pseudomonic acid mixture, mupirocin, which is used clinically against MRSA, and the pyrrothine core of holomycin. Thiomarinols are a novel family of natural compounds with potent antimicrobial activity. Understanding how complex antibiotics are synthesized by their producer bacteria to create new families of bioactive compounds [33].

Recently, the violacein produced by *Chromobacterium violaceum* has an inhibitory effect against *S. aureus* isolated from bovine mastitis and displays synergism with penicillin [20].

The F3 compound studied in this work is effective against *Xanthomonas citri* pv. Citri, which causes citrus canker lesions [22]. The F3 fraction was initially tested against other bacteria such as *Staphylococcus* spp., *Enterococcus* spp., *Klebsiella pneumoniae*, *E. coli*, *P. aeruginosa* and *Salmonella enterica* serovar Typhimurium and Enteritidis. The initial results demonstrated that the compound was effective against *S. aureus*, *S. epidermidis*, *Enterococcus faecium* and *K. pneumoniae* (data not shown). Among these bacteria, *Staphylococcus* spp. had the largest inhibition zones in the diffusion disc test.

Because many hospital infections involve MRSA, we evaluated the effect of the F3 fraction standard and clinical strains on MRSA.

The zones of inhibition in the diffusion disc test and the MICs for the F3 fraction were similar among the standard and clinical MRSA and MSSA strains. The MICs for MRSA were higher than for *X. citri*; however, these results are very significant and important for therapies used to treat diseases caused by these strains. For the experiments discussed, the F3d fraction was purified from the F3 fraction.

When we evaluated the F3d effect, we found that within a few hours (2 to 5 h), the number of CFUs decreased significantly, indicating that this compound acts rapidly on these strains. In our *in vitro* tests, the F3d fraction had a bactericidal effect at 200 μg/ml.

By electron microscopy, we observed cellular morphological alterations within a few hours of incubation with lower concentrations of the F3d fraction. The alterations were similar to the effects on *X. citri*[22]. In addition to reducing of the number of CFUs, we observed deformation and sinking of the bacterial cell wall (Figure 3C and 3D).

The results of the cytotoxicity assay demonstrated that the F3d compound does not have cytopathic effects and is not cytotoxic to LLC-MK_2_ cells, suggesting low toxicity to the host. Thus, this compound could be used in patients, without side-effects.

**Figure 4:**
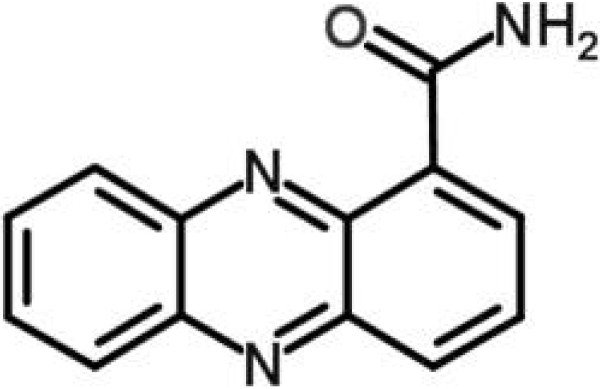
Chemical structure of phenazine-1-carboxamide.

Phenazines are natural products found in *Pseudomonas* spp., *Streptomyces* and a few other genera from soil or marine habitats. Phenazines are large family of colorful, nitrogen-containing tricyclic molecules with antibiotic, antitumor, and antiparasitic activities [34]. Phenazines isolated from *Pseudomonas* species (e.g. *aeruginosa*, *aureofaciens*, *fluorescens* and *cepacia*) are mostly simple hydroxyl- and carboxyl-substituted structures. Pyocyanin (5-N-methyl-1-hydroxyphenazine), phenazine-1-carboxylic (PCA) and phenazine-1-carboxamide (Figure 4) are among the phenazines produced by Pseudomonads, mainly rhizosphere isolates [35]. In our study, we identified phenazine-1-carboxamide (Figure 4) in the F3d fraction. This substance was effective against *S. aureus*, including the MRSA strain N315. The MIC (250 μg/ml) of phenazine for these strains was greater than for the F3 and F3d fraction (125 μg/ml) (Table 1). In other words, the phenazine was less effective than the F3 and F3d compounds. The growth and viability curve with phenazine was similar to F3 and F3d treatment (Figure 2). These results demonstrate that phenazine-1-carboxamide from the F3d fraction has a slight inhibitory effect on *S. aureus*, including MRSA. The higher MIC of phenazine suggests that the F3d fraction contains other inhibitory substances or synergistic compounds. Physiologically, phenazine physiological inhibits and controls nucleic acid and protein synthesis [34]. Therefore, the modes of action for phenazines may include interactions with DNA (intercalation or groove binding), topoisomerases, anti-oxidants or charge-transferring molecules [34].

Some studies have demonstrated that phenazine efficiently inhibits the growth of bacterial and fungi [36,37]. There are no studies showing that phenazine-1-carboxamide has an antibacterial effect on MRSA. Another study has reported that phenazine has antimicrobial activity on major rice pathogens, such as *Rhizoctonia solani* and *Xanthomonas oryzae* pv. Oryzae [38]. Ecological investigations of the action and crucial role of phenazines have focused in suppressing fungal pathogens of plants such as *Fusarium oxysporum* and *Gaeumannomyces graminis*[39].

The combination of phenazine-1-carboxamide and silver nanoparticle produced by *F. oxysporum* showed synergic effect decreasing up to 32 times the MIC value of phenazine. Studies involving synergism have been very important for antibacterial therapy mainly for multiresistant bacteria. Some natural products have shown anti-staphylococcal activities weaker than others antibiotics, but synergic interactions may use different mechanism of action or pathways to demonstrate their antimicrobial effects, as resulting in the lowered MICs [40]. The combination of current antibiotics with plant-derived antibacterial agents has showed synergic effect against MRSA [31].

An organohalogen compound also was identified from F3d fraction and showed high inhibitory activity against MRSA strain N315. The specific structure has not been identified, but future studies on this organohalogen will conducted. Like penicillin, morphine, vincristine, aspirin and other natural products, several natural organohalogens have important medicinal value [41,42]. The 2,4-dibromophenol-6-chloro compound isolated from a marine filamentous bacterium, *Pseudoalteromonas luteoviolacea*, shows antibacterial activity against MRSA [43].

There are few effective antimicrobials against multiresistant bacteria including MRSA strains. These antimicrobials are often associated with high costs and serious side effects for the patients. Many different natural antimicrobials have been studied as an alternative to control these infections. Our study suggests that the use of a secondary metabolite from bacteria such as *P. aeruginosa* could be effective again MRSA strains that cause diseases in humans and other animals. This compound may be a good alternative to treat and control of infections caused by MRSA.

## Competing interest

The authors declared that they have no competing interests.

## Authors’ contributions

VC performed all experiments (master thesis). AO purified and provided phenazine-carboxamide. EN assisted synergism and electronic microscopy assays. MP provided MRSA clinical strains and involved in analysis of clinical aspects. CA supervised electronic microscopy. WS involved in drug interaction analysis. ND involved in silver nanoparticles. GA involved in compounds of *Pseudomonas aeruginosa* (F3 and F3d fractions). RK involved in antibacterial activity. GN involved in designing of the project, results analysis, and write up of the manuscript. All authors read and approved the final manuscript.
